# A qualitative study to explore the acceptability and feasibility of implementing person-focused evidence-based pain education concepts in pre-registration physiotherapy training

**DOI:** 10.3389/fpain.2023.1162387

**Published:** 2023-04-11

**Authors:** Kate Thompson, James Milligan, Michelle Briggs, Janet A. Deane, Mark I. Johnson

**Affiliations:** ^1^ Centre for Pain Research, School of Health, Leeds Beckett University, Leeds, United Kingdom; ^2^ Clinical Professor of Nursing & Director, Manchester Clinical Academic Centre (MCAC) for Nurses, Midwives and AHPs (NMAHPs), Manchester University NHS Foundation Trust (MFT) & School of Health Sciences, The University of Manchester, Manchester, United Kingdom; ^3^ Research and Innovation Division, Manchester University Hospitals NHS Foundation Trust, Manchester, United Kingdom; ^4^ Centre of Precision Rehabilitation for Spinal Pain, School of Sport, Exercise & Rehabilitation Sciences, University of Birmingham, Birmingham, United Kingdom

**Keywords:** pain education, pain, physical therapy, physiotherapy, interprofessional and communication skills, person centered

## Abstract

**Objectives:**

The purpose of this study was to engage with physiotherapy clinicians, academics, physiotherapy students and patients to explore the acceptability, feasibility, and practical considerations of implementing person-focused evidence-based pain education concepts, identified from our previous research, in pre-registration physiotherapy training.

**Design:**

This qualitative study took a person-focused approach to ground pain education in the perspectives and experiences of people who deliver and use it. Data was collected *via* focus groups and in-depth semi-structured interviews. Data was analysed using the seven stage Framework approach.

**Setting:**

Focus groups and interviews were conducted either face to face, *via* video conferencing or *via* telephone. This depended on geographical location, participant preference, and towards the end of data collection the limitations on in-person contact due to the Covid-19 pandemic.

**Participants:**

UK based physiotherapy clinicians, physiotherapy students, academics and patients living with pain were purposively sampled and invited to take part.

**Results:**

Five focus groups and six semi-structured interviews were conducted with twenty-nine participants. Four key dimensions evolved from the dataset that encapsulate concepts underpinning the acceptability and feasibility of implementing pain education in pre-registration physiotherapy training. These are (1) make pain education authentic to reflect diverse, *real* patient scenarios, (2) demonstrate the value that pain education adds, (3) be creative by engaging students with content that requires active participation, (4) openly discuss the challenges and embrace scope of practice.

**Conclusions:**

These key dimensions shift the focus of pain education towards practically engaging content that reflects people experiencing pain from diverse sociocultural backgrounds. This study highlights the need for creativity in curriculum design and the importance of preparing graduates for the challenges that they will face in clinical practice.

## Introduction

Chronic pain impacts negatively on the lives of individuals and causes a burden on health and social care systems globally. Estimates of the prevalence of chronic pain suggest that over 40% of adults experience pain on a daily basis and that over 10% of adults find this pain debilitating (1–3). Often, people experiencing pain seek support from physiotherapists who require an understanding of the multi-dimensional nature of pain and a broad skill set to manage the variety of pain conditions presenting in clinic. The foundation of knowledge and skill acquisition about pain is established in pre-registration training.

Pain education in pre-registration training is variable and, in some cases, inadequate (4–7). Historically, pain education has focused on biomedical concepts rather than practical skills (8), referred to by the International Association for the Study of Pain (IASP) as the “theory-practice gap” (9). Guidance documents for pain education of health care professionals have been published to address this theory-practice gap including core competencies (10), pain curricula (11), and practical guides for pain education (12), and these documents have been used to inform the design of pre-registration training. However, the complexities of implementing pain education in pre-registration training requires more investigation.

Previously, we evaluated pain education through a complex intervention lens by synthesising information about pain education contained in published research, policy, curricula, competencies, frameworks and the views of people experiencing pain (8, 13). Concepts emerged in relation to the context, content, delivery, and outcome of pain education as summarised in Table 1. The acceptability and feasibility of implementing pain education aligned to these concepts is unknown. Therefore, the purpose of the present study was to engage with key stakeholders (i.e., physiotherapy clinicians and academics, physiotherapy students and patients) to explore their views about the acceptability, feasibility, and practical considerations of implementing these concepts, identified by our previous research, in pre-registration physiotherapy training. It was decided that engaging stakeholders using qualitative methods would add personal and contextual experiences about the lived experience of pain, pain management, and pain education, that would inform the development of authentic educational strategies that reflected the clinical environment.

**Table 1:** Person-focused evidence-based pain education concepts.

Context	Content
**Concepts** • Provide context by introducing students to patients’ needs when experiencing pain • Map learning activities to patients needs **Example** Patients’ needs are complex when experiencing pain. Patients’ needs include: – “To feel listened to and believed” – “A reciprocal consultation” – “To understand the meaning of pain” – “To understand the mind-body link” – “Accessible and realistic pain management” – “Hope and direction from a professional”	**Concepts** • Develop pain management skills • Underpin with contemporary pain science • Learn to assess and challenge [own and others] attitudes and beliefs about pain **Example** – Develop active listening & communication skills – Practice explaining pain – Practice difficult conversations around the origins and meaning of pain – Learn to co-create goals, outcomes and management plans – Learn to teach, motivate, coach and give feedback – Embed IASP core curricula
Delivery	Outcome
**Concepts** • Include all stakeholders in pain education delivery • Move delivery away from theoretical towards practically engaging activities **Example** – Practice pain assessment and management with patients/actors – Use technology e.g., virtual reality to experience ‘real’ scenarios – Include current clinical expertise to provide case studies and scenarios – Engage final year students in first year education – Learn in multi-disciplinary groups	**Concepts** • Evaluate confidence and competence in pain assessment and pain management using a competency-based approach • Demonstrate & evaluate the impact of learning relative to patients needs **Example** – Introduce pain education competencies & outcomes (e.g., IASP endorsed competencies/PPA framework) to evaluate the impact of pain education – Include patients and clinicians in evaluating the outcome and impact of learning – Map learning outcomes to clinical placements including patient outcomes

IASP, International Assocition for the Study of Pain.

## Materials and methods

### Design

This qualitative study took a pragmatic person-focused approach to ground pain education in the perspectives and experiences of people who deliver and use it (14). Focus groups and semi-structured interviews were conducted using Framework analysis (15). The conduct of the study was guided by the COnsolidated criteria for REporting Qualitative research (COREQ) (16).

### Sampling strategy

Physiotherapy clinicians, physiotherapy students, physiotherapy academics and patients living with pain were purposively sampled and invited to take part in a focus group discussion or an in-depth semi-structured interview.

A strategic and targeted approach was taken by inviting clinicians and academics active in the field of pain education. Patients were approached *via* gatekeepers of existing service user and carer groups, pain charities, pain support groups and social media. Physiotherapy students enrolled on a UK pre-registration physiotherapy programme and who had completed at least 3 clinical placements were approached *via* gatekeepers (course leaders/course directors) of their respective programmes of study (courses). The study was also advertised *via* professional networks, university networks social media and pain charities. A snowball effect occurred where the advert was forwarded and shared with those who may meet the eligibility criteria. Volunteers contacted the principal investigator (KT), were provided with a participant information sheet, and were invited to a study visit where informed consent to participate was gained.

### Data collection

Focus groups and interviews were conducted by one author (KT) either face to face, *via* video conferencing or *via* telephone. This depended on geographical location, participant preference, and towards the end of data collection the limitations on in-person contact due to the Covid-19 pandemic. Focus groups and interviews were recorded and transcribed verbatim. Transcripts were uploaded and analysed in NVivo (17). Field notes were taken where possible e.g., tone of voice, gesturing, animated response to questions. In the focus groups and interviews, participants were presented with person-focused evidence-based pain education concepts identified by our previous research (8, 13). Participants were asked about

• their experiences, attitudes and beliefs about pain education
• their views and opinions about the acceptability (including appropriateness, suitability, likes and dislikes) of our person-focused evidence-based pain education concepts
• their views on the feasibility (including strengths, opportunities, barriers, challenges, and limitations) of implementing our person-focused evidence-based pain education concepts in preregistration physiotherapy training

The goal was to conduct 6–9 focus groups or equivalent interviews, or until there was evidence of data saturation.

### Pilot

Concepts for pain education, identified by our previous research, were printed and presented in-person to a pilot group that included a physiotherapy clinician, physiotherapy academic, person with experience of pain and physiotherapy student prior to data collection. The purpose of this pilot group was to explore how best to present our person-focused evidence-based pain education concepts to study participants with diverse experiences and health literacy. KT facilitated discussions about the nature of the key concepts, potential questions and structure for the focus groups and interviews, and the format to present the key concepts. No major concerns were raised by participants during the pilot and only minor amendments were made to the language and content of documents and to the timing of the interview schedule to maximise discussion and debate.

The final format of delivery of the concepts used in the focus groups and interviews is provided in Table 1. Supplementary File S1 provides the semi-structured interview guide.

### Data analysis

Data was analysed using the seven stage Framework approach (15, 18) which included: transcription, familiarisation, labelling, indexing, sorting, charting data and abstracting key dimensions. Data was analysed using both inductive and deductive themes. Deductively, we specifically wanted to gather views about the acceptability of the concepts that participants were presented with, (including appropriateness, suitability, likes and dislikes) Data was labelled deductively as “likes” or “dislikes”. We also wanted to gather their views about the feasibility (practicalities) of implementing the concepts in preregistration pain education. Data was labelled deductively as “strengths and opportunities” and “barriers and limitations”. Data that did not fit into a pre-existing theme was labelled inductively. Data analysis was managed using NVivo (17).

To add rigour and to mitigate the perspective of one researcher dominating, two researchers (KT & JD) independently labelled three transcripts, one from each stakeholder group. The labels were discussed to construct an initial thematic framework (set of labels) which were subsequently applied to all remaining transcripts. Data that did not fit within one of the existing labels was given a new inductive label until all data was indexed and sorted into themes.

Once all data had been labelled, one author (KT) wrote a precis descriptive summary for each theme. Data analysis went beyond description to explore key dimensions that underpinned the acceptability and feasibility of implementing our person-focused evidence-based pain education concepts in pre-registration physiotherapy training (18).

## Results

### Description of participants

Five focus groups and six semi-structured interviews were conducted with twenty-nine participants (seventeen females and twelve males). Two focus groups were conducted with physiotherapy students (four females and four males) and three focus groups conducted with a mixture of physiotherapy clinicians and physiotherapy academics (seven females and eight males). In addition, one female physiotherapy clinical academic was interviewed separately as they were unable to attend a focus group. Five one-to-one interviews were undertaken with patients (five females). It was not possible to achieve the original target to conduct six to nine focus groups with equivalent numbers of participants for one-to-one semi-structured interviews because recruitment had to be closed due to restrictions imposed by the outbreak of Covid-19. Nevertheless, we exceeded minimal threshold for recruitment and data saturation was evident.

The purposeful approach to recruitment resulted in a sample of participants with diverse experiences ensuring “equal voice” across stakeholder groups. All patients had experienced physiotherapy for musculoskeletal pain. Pain duration ranged from 1 year to over 40 years. All physiotherapy students had completed at least three clinical placements with experience of assessing and supporting people living with pain. Clinicians and clinical academics had diverse experiences and included full time clinical NHS and private sector roles, mixed practitioner/educator roles, professional advisors, specialists in pain management, and academics working at different levels, including early career to senior academics.

### Framework analysis

We interrogated the data transcripts within and across the different stakeholder groups. Four key themes emerged which are articulated as “key dimensions”. This is in keeping with Framework methodology as the final output of the analysis of the whole dataset (18). In Framework methodology, the purpose of displaying the qualitative data in a matrix is to summarise, display and organise the data. A “key dimension” was interpreted to be “key” if it captured data about the acceptability and feasibility of implementing the pain education concepts across all stakeholder groups (18, 19). An example of data analysis is provided in Supplementary File S2. A description of each of the abstracted key dimensions are presented below.

### DIMENSION 1: Make pain education authentic to reflect diverse, *real* patient scenarios

Participants frequently talked about the importance of pain education being “real” to ensure education incorporates patient scenarios that reflect diverse clinical practice. Encapsulating authenticity, it was important to participants that students engage in content that provides a window into the real lives of people who are experiencing pain.

*[X] I think there's nothing like practice and there's nothing like having real people, in other words, real patients talking. [PARTICIPANT 27: PATIENT]*

*[X] I really do think the value of getting people with lived experience of pain to talk about what's going on, I think that's what they would value, what the people with the lived experience have valued and would value. [PARTICIPANT 21: PHYSIOTHERAPIST]*

Reflecting on their own experiences, participants liked the fact that many of the concepts emphasised activities that were grounded in “real life”, believing that this would facilitate the development of skills needed for clinical practice.

*[X] I think it's important from an empathy perspective, to try and bring in the patient as much as possible. So I think if you can bring in patients or if you can have patient voice in videos etc., that definitely helps. [PARTICIPANT 26: PATIENT]*

This included using virtual or simulated patients as a tool for students to be immersed in clinical scenarios.

*[X] I think there's lots of good things there and I'm just looking at simulated patients and practicing and real scenarios—those kind of things I think are really important. [PARTICIPANT 24: PATIENT]*

Participants offered suggestions of how pain education tools that reflect patients' lives could be developed and implemented to improve the skills of student physiotherapists. Participants wanted students to appreciate the wide-ranging impact that pain has on individuals' lives by learning to actively listen to a person's narrative and to actively seek a full understanding of a patient's experiences.

*[X] I think that idea of ‘patient's story’ is really important, to adapt to it as well as just listening. I think the needs are there, I think they're all fine, but I think also about, the patient's actual full narrative that goes along with that. [PARTICIPANT 21: PHYSIOTHERAPIST]*

Participants believed that it was imperative to embed “real life” clinical scenarios early in pre-registration training to develop and practice conversational skills to support a *person* experiencing pain.

*[X] I like the practical element of it. The more interactive work the better, the more time they [physiotherapy students] go away and engage with the materials themselves, actively, the better. [PARTICIPANT 17: PHYSIOTHERAPIST]*

*[X] The other bits on the practice [are] difficult conversations and goal setting, I think are imperative. Practicing that difficult conversation has got to start early, definitely. [PARTICIPANT 16: PHYSIOTHERAPIST]*

*[X] I think the more practice people [physiotherapy students] get of verbalising these very complex explanations, sometimes complex biology, complex psychology, complex sociology, that the easier it becomes for people, so that practical approach is very important. [PARTICIPANT 26: PATIENT]*

### DIMENSION 2: Demonstrate the value that pain education adds

This dimension reflects data about the *value* that pain education adds to pre-registration training, that was not always necessarily explicit or overt. For instance, participants believed that embedding person-focused pain education has the potential to add value by facilitating and engaging students in wider conversations about individuals and society, to develop holistic health professionals who appreciate diverse sociocultural factors in health and wellbeing. In this respect, one participant described this approach to pain education as the perfect “starter topic” to physiotherapy training.

*[X] I actually think that pain is sort of probably a really good vehicle to incorporate straight away. Hit them with it early as a real vehicle to see people as that, as thinking, feeling beings that exist in a real life world.. I think it's a really good springboard to the rest of their training [PARTICIPANT 14: PHYSIOTHERAPIST]*

Participants reflected on their own experiences of pain education discussing the added value that they perceived to have got from their training.

*[X] We just used to meet for coffee first thing, we did nothing else, we just used to discuss where we'd seen aspects of pain in society and life in the papers, on the news, in all sorts of media and that used to generate fantastic discussion. [PARTICIPANT 12: PHYSIOTHERAPIST]*

*[X] Chronic pain is associated with a whole host of other lifestyle and health and wellbeing factors, so part of a health and wellbeing module, where we talk about exercise and nutrition and other lifestyle factors. [PARTICIPANT 2: PHYSIOTHERAPY STUDENT]*

Participants reflected on the value of developing skills to be able to hold meaningful conversations with people, in considering differing levels of health literacy, language and conversation skills.

*[X] I do think depending on the person that you deal with, some people are happy to have more information and to understand the processes and things of the pain, of why it operates and how it happens. I'm not saying that's right for everybody, but there will be that level of person that's important to understand why and how, which then gives them the sort of understanding to move forward with what they're trying to do. [PARTICIPANT 22: PATIENT]*

*[X] Someone mentioned before about the context of society and culture, you know, it's completely embedded within that and embodies within the person and I think that, when you can get the student to appreciate that and some general principles and to understand themselves and to look after themselves and where they're coming from, their own biases, it is a start point [PARTICIPANT 11: PHYSIOTHERAPIST]*

### DIMENSION 3: Be creative—engage students with content that requires active participation

This key dimension was abstracted from detected data about creating learning activities that students find interactive, engaging and memorable. Data analysis revealed significant coverage of discussions relating to the use of simulation in pain education. Physiotherapy students discussed that the use of simulation could create “safe spaces” to practice pain assessment and pain management prior to patient-facing clinical placements.

*[X] I really like this bit.. using patients/actors, because in our neuro modules we had somebody come in and talking about their stroke experience and it was really nice to just listen to somebody, when you're not being assessed or you're not having to ask the questions but just listening to their story [PARTICIPANT 3: STUDENT]*

Participants reported that simulation gave them the opportunity to interact and experience some of the feelings that occur in real patient scenarios.

*[X] But that whole situation when you're in there, it's quite, oh, like is this person actually, do I actually need to do stuff with them, because they are really good, they just put you in that mind frame [PARTICIPANT 2: STUDENT]*

Participants believed that the use of technology would be positive for pain education because it aligns with students' worlds and has the potential to make learning about pain more engaging and exciting.

*[X] The support some of those other emerging technologies can give us in this sort of type of teaching and approach to people I think is really exciting. That's what they [students] interact with all the time, you know, electronic game sites being problem-based thinking when they're playing games. [PARTICIPANT 14: CLINICAL ACADEMIC]*

*[X] I think [using technology] students will have a massive advantage going forwards, confidence wise, going forwards [PARTICIPANT 26: PATIENT]*

*[X] I think if we can employ some of this technology, that sounds really exciting what you're talking about there. [PARTICIPANT 15: CLINICAL ACADEMIC]*

Participants reflected on their learning experiences, describing a dislike for the prospect of pain education being any more “theoretical” than they had experienced. Participants believed that theoretical understanding of pain science can be gained by independent directed learning and that a better use of face-to-face learning is to gain practical skills required to assess and support people in pain. Participants believed that curricula should foster active rather than passive learning, for instance, using approaches such as problem-based learning.

*[X] Perhaps P.B.L. (problem based learning) was one of the best sort of vehicles, so very much that student engagement, course discipline learning, which actually really makes a massive impact [PARTICIPANT 14: CLINICAL ACADEMIC]*

*[X] I think if we got more of a focus on those personable skills and those things in there that would help with how we communicate.. pain management has to come into it and just giving us the tools to be that all-rounded practitioner. [PARTICIPANT 2: STUDENT]*

There were mixed views on the prospect of actors or simulated patients to create authentic patient scenarios. Some participants believed that actors could never portray what it is like to really have chronic pain. Interestingly, participants reported that virtual patients could be viewed as more authentic than actors because virtual patients can be based upon and created from *real* patient scenarios and therefore would not be “acting”.

*[X] I just don't think that the responses you get from actors or simulated patients are anything like what you genuinely get. [PARTICIPANT 16: PHYSIOTHERAPIST]*

*[X] I think I'd be a little reluctant to have actors and simulated patients, much more try and get video with patients and include that kind of assessment. [PARTICIPANT 18: CLINICAL EDUCATOR]*

*[X] I think what you are doing looks to be some very good stuff. The only thing I'm not sure about is actors for the reasons I've already told you. Its acting—it's not what it's really like [PARTICIPANT 23: PATIENT]*

### DIMENSION 4: Openly discuss the challenges and embrace scope of practice

This key dimension was abstracted from data about the challenges of implementing pain education in pre-registration physiotherapy training and in clinical practice. Participants identified barriers and threats to the delivery of pain education. Participants believed that some of the challenges include working alongside qualified physiotherapists who do not embrace contemporary pain management, lack of guidance from regulatory bodies and time constraints related to “business like” clinical models of care.

*[X] I'm just conscious of the fact that they're (students) sort of plunged into departments with huge waiting lists and pressure to do everything in three appointments, and a lot of fairly mechanical, bio-medical outcome measures. You know what I mean? I'm just a bit concerned that, you know, you might end up with really rather unhappy people. [PARTICIPANT 21: PHYSIOTHERAPIST]*

There was a sense that for pre-registration pain education to succeed, some areas of clinical practice needed to be challenged. For example, when participants reflected on current physiotherapy services or physiotherapy attitudes that were perceived to negatively impact students' learning.

*[X] I've been with some educators and qualified physios and they've not followed this kind of concept of pain management. They dismissed it and then obviously you [the student] is following, to a degree, this qualified physiotherapist's actions [PARTICIPANT 6: PHYSIOTHERAPY STUDENT]*

*[X] I think it's a culture thing [physiotherapy profession culture]. If it's only becoming a thing now, then the people who are educating us [placement educators] wouldn't have had this education. [PARTICIPANT 7: PHYSIOTHERAPY STUDENT]*

Participants perceived a need to better prepare students for the challenges of modern clinical practice by raising awareness of professional culture and developing appropriate skills to cope with and challenge outdated views and clinical constraints.

*[X] For me, the stumbling block is that it takes time for the things that are needed [for pain education]—to discuss things and to plan things and of course that's a great financial luxury, isn't it? [PARTICIPANT 24: PATIENT]*

*[X] In MSK (musculoskeletal outpatients) we work in half hour appointments. It's not the best way of treating chronic pain patients, we need more time [with students] and resources [PARTICIPANT 8: PHYSIOTHERAPIST]*

Scope of practice was frequently mentioned by participants, often with contradictory views. Some participants believed that physiotherapists need to embrace their role in assessing and supporting people with psychological and social components of pain, whilst others felt this was beyond physiotherapists scope of practice and that physiotherapists should make better use of the multi-disciplinary team.

*[X] It's much easier actually to put your hands on a patient and press it better or give them ten repetitions of X, Y or Z than it is to listen to somebody and the only other thing I would say is that I think the scope of practice comes in a bit. I mean I personally think that it is well within our scope of practice to do anything which is, talk about anything which is impacting the patients pain directly [PARTICIPANT 21: PHYSIOTHERAPIST]*

*[X] I think you need to have quite clear pathways for additional support, particularly nowadays when people can start disclosing stuff which can be quite distressing and which obviously does take you then really outside of your scope of practice, particularly if you’re a student or young clinician. We’re not psychologists. [PARTICIPANT 21: PHYSIOTHERAPIST]*

## Discussion

In this qualitative study we used Framework analysis to search for key dimensions that underpin the acceptability and feasibility of implementing our person focused pain education concepts in preregistration physiotherapy training. Four key dimensions emerged that encapsulate data across the stakeholder groups. We argue that to successfully embed person-focused evidence-based pain education in preregistration physiotherapy training, educators need to ensure that pain education:

(1) is *authentic* to reflect the diversity of real-life patient scenarios,
(2) explains the *value* that person-focused evidence-based pain education adds,
(3) is *creative* in design to engage students with content through active participation,
(4) *openly discuss*es the challenges and embraces scope of practice.

Implementing these key dimensions within clinical and education settings will be discussed, relative to other literature, following an appraisal of the strengths and limitations of the study.

### Strengths and limitations

This qualitative study had several strengths. The views and experiences of multiple stakeholders were sought giving equal voice to patients, physiotherapy students, clinicians and academics. Our recruitment strategy allowed participants to select their preferred method of data capture (i.e., focus group or one-to-one interview) and this promoted inclusivity so that the voice of people from diverse backgrounds and stakeholder groups was captured. Refinement of methodology following pilot focus groups ensured the robustness of interview technique and data collection and analysis. Transcripts were independently coded by two authors and agreement reached through discussions with the full authorship team.

The main limitation of the study was variations in data recording procedures. Field notes taken during in-person focus groups and interviews were more comprehensive and allowed observations of physical responses to questions than telephone interviews. Thus, there was more depth to the data collected from in-person sessions. All patient participants opted for a one-to-one interview over a focus group discussion. We do not know the reason for this, although we speculate that patients may have been reluctant to disclose personal thoughts and feelings in the presence of others. We did not directly invite health policy makers or health regulators and their inclusion may have added more breadth to the data analysis. Nevertheless, we did capture the views of participants who worked in advisory roles for national and international organisations such as the British Pain Society and the Health and Care Professions Council.

### Implications for clinical and educational settings

In our key dimensions, we advocate implementing pain education that is authentic, that reflects diverse and real patient scenarios and that demonstrates its value. To successfully implement pain education that is guided by these key dimensions there is a requirement that educators recognise, include and respond to a range of dimensions of diversity that represent people from varied backgrounds with different experiences. For instance, ensuring that patient scenarios represent people with different experiences of healthcare, sociocultural values, socioeconomic determinants of health and belief systems about pain. This is important, to prepare graduates to work in health systems that are fit for purpose, particularly in the landscape of health inequalities in chronic pain (20). There is little published research to evaluate the impact and delivery of person-focussed pain education for pre-registration physiotherapy training. However, O'Shaughnessy and Tilki proposed a model for “cultural competence” for physiotherapists (21). The emphasis of the training was to enable staff to explore their own values, beliefs and ideas relative to cultural competence. There may be learning that can be applied to pain education in pre-registration training. Furthermore, information regarding wider socioeconomic determinants and disparities in chronic pain can be used to inform the development of authentic pain education materials that reflect diverse and real patient scenarios (20, 22, 23).

In our key dimensions we advocate engaging students with content that requires active participation. This requires consideration of operational logistics especially with known challenges such as limited time and space, and high demands to cover many topics in pre-registration curricula. Ensuring that students have had sufficient theoretical content to support the development of their practical skills is challenging. One solution could be the use of a “flipped classroom” which involves students engaging in preparatory theoretical content in advance of classroom-based learning. This prioritises classroom learning that focusses on skills development through authentic, valuable, creative, and interactive learning opportunities, such as patient scenarios, in which to apply theoretical concepts (24). There is a paucity of research to evaluate the use of a flipped classroom approach for pain education in pre-registration physiotherapy. However, Røe et al. (25) reported greater improvements in student outcomes following a flipped classroom approach for skills and knowledge of musculoskeletal physiotherapy compared with conventional teaching. Evaluations of flipped learning in medical education demonstrate increased motivation and engagement (26) and improvements in affective and soft skills (27). Limitations to flipped classrooms include not adequately preparing for in-classroom sessions, lack of access to tutors/resources and students not working optimally in classroom activities (25, 28). These are important considerations when planning pain education content that requires active participation.

Finally, in our key dimensions we advocate openly discussing the challenges and embracing scope of physiotherapy practice in pre-registration pain education, to develop graduates who can positively influence and impact pain management, particularly in clinical services that may be practicing more biomedical approaches. However, we acknowledge that this is challenging, particularly for newly graduated physiotherapists, who would need the confidence and credibility to promote change. In the U.K., the Chartered Society of Physiotherapy have previously run a series of events and publications to raise the profile of leadership within the physiotherapy profession, arguing that leadership is as important for student and graduate physiotherapists as those in strategic and managerial positions (29). Incorporating and nurturing leadership skills early in physiotherapy training will help to equip students and graduates with the skills to discuss the challenges of pain management and physiotherapy scope of practice; and to advocate change towards more contemporary models of assessing, treating and caring for patients presenting with pain.

### Future directions

There is a need to build an evidence base for pre-registration pain education, especially from the perspectives of people who experience pain. The four key dimensions emerging from our analysis of the views of stakeholders can be used to guide the design and implementation of person-focused evidence-based pain education curricula and materials. We recommend that any future pain educational materials aligned with these four key dimensions be shared with the wider pain education community so that their impact can be evaluated over several domains such as student learning, placement and patient outcomes. We advocate the use of qualitative, mixed methods or case study methodologies for such evaluations.

## Conclusion

Our study engaged with various stakeholders, including patients, to explore the acceptability, feasibility and implementation of our person-focused evidence-based pain education concepts in pre-registration physiotherapy training. Emerging themes were to make pain education authentic and real-life, emphasising the value of the person not just the pain, and utilises creative and participatory learning opportunities that reveal the challenges and scope of clinical practice. In conclusion, we argue that the focus of pain education needs to shift towards the realities of clinical practice by creating content and learning opportunities that represent people experiencing various types of pain from diverse sociocultural backgrounds. This will involve creativity in curriculum design including, for example, use of flipped classroom contexts and simulated clinical scenarios using modern technologies. This will enable students to develop necessary skills and knowledge in safe learning environments so that they become confident and competent to embrace the challenges that they may face in clinical practice.

## Data Availability

The original contributions presented in the study are included in the article/**Supplementary Material**, further inquiries can be directed to the corresponding authors.
