# Supplementary material for: A qualitative study to explore the acceptability and feasibility of implementing person-focused evidence-based pain education concepts in pre-registration physiotherapy training
Source: Front Pain Res (Lausanne). 2023 Apr 11;4:1162387. doi: 10.3389/fpain.2023.1162387 (PMC10126772; doi:10.3389/fpain.2023.1162387)
Supplement: Supplementary file 1 [file Table1.docx]

# Supplementary file 1: Topic Guide for qualitative focus groups and interviews

**Introduction**

- Introduce the researcher
- Introduce the study topic and funder (Health Education England)
- Explain the aims and objectives of the study
- Explain confidentiality and anonymity
- Explain recording (audio-recorded) and length (1-1.5 hours), nature of discussion, outputs and data storage
- Check if participants have any questions
- Check that participants are happy to continue

**Background**

Aim to get participants talking about their experiences of pain and or pain education and to explore the context of their current circumstances.

- Describe current role/situation
- Describe experience of pain or pain management

AND/OR

- Describe experience of delivering pain education (pedagogic/assessment approaches, barriers, successes)

AND/OR

- Describe experience of receiving pain education (experience of teaching, learning and assessment methods)

**Core discussion**

- Introduce the person-focussed evidence-based pain education concepts
- Define concepts and meanings
- Enable participants to think, reflect and ask questions
- Encourage participants to reflect on their current practice, experiences or beliefs about pain and/or pain education
- Draw out views, attitudes, opinions and priorities – are the concepts acceptable, feasible, and practical
- Could you implement it in practice? If so, how? If not, why not.
- What are the issues with it?
- What is missing/ how would you improve it?
- Where possible root discussions in specific examples and cases

**Wind down and summary**

- Questions and suggestions for the future
- Has anything been missed or not covered?
- Are there any other points that participants would like to raise?

**Conclusion**

- Re-iterate confidentiality
