# Supplementary material for: A qualitative study to explore the acceptability and feasibility of implementing person-focused evidence-based pain education concepts in pre-registration physiotherapy training
Source: Front Pain Res (Lausanne). 2023 Apr 11;4:1162387. doi: 10.3389/fpain.2023.1162387 (PMC10126772; doi:10.3389/fpain.2023.1162387)
Supplement: Supplementary file 2 [file Table2.docx]

| **Example Framework analysis** | | | |
| --- | --- | --- | --- |
| 1. Raw data | 1. Data summary & display (summarising whilst retaining some raw data) | 1. Detected elements (interpreting key elements) | 1. Key dimensions (bringing key elements together) |
| [X] I like the idea of assessing pain and I like the use of the IASP competencies, the frameworks, they already exist, we don’t need to re-invent the wheel and my other thought was in the blue section, stakeholders, it is a great idea definitely to involve the stakeholders.  [X] I like that you’ve said we can get students involved in the delivery of pain education, I don’t think that’s something that at xxx we utilised a lot, any thoughts anyone?  [X] Perhaps P.B.L. is one of the best sort of vehicles, so very much that student engagement, course discipline learning, which actually really makes a massive impact  [X] I think the more practice people get of verbalising these very complex explanations, sometimes complex biology, complex psychology, complex sociology, that the easier it becomes for people, so that practical approach is very important.  [X] I could probably look through our different curricula and identify where we teach all of these, or some of these different aspects, but I think the unique thing and the really good thing about this is bringing it together  [X] That’s a really relevant point, I think a lot of people said, I don’t know how to deliver it, I know it but I don’t know how to deliver it. This is an opportunity to do that and I also agree with *** that together would be really key.  [X] There’s that assessment of it at the end and specifically the assessment of it being almost, I think a key bit of that is some sort of behavioural assessment aspect too, that it’s not just knowledge and understanding but it’s, you know, it’s not just psycho-motor skills but also the ability to, you know, to do this in practice I think is key.  [X] I would even say that the virtual reality patient scenarios are probably more real life than getting some students to try to act that out. That’s the feedback that we got as well is that, you know, it’s very, very difficult to become a patient, very.  [X] I think actually the virtual patient potentially might work better because if the actors already bought in, you know, I’m not sure that they always communicate as well, or would create quite such a realistic experience for the students, I think that’s quite an interesting idea.  [X] The support some of those other emerging technologies can give us in this sort of type of teaching and approach to people I think is really exciting and I would probably historically be one of those people saying, oh you can’t get a traditional machine to mimic a person and we’ve got to forget about that you know, because it’ll make people think that all humans are computers, and I think this thing is really exciting because it overcomes a lot of those objections .. (talking about young people) .. that’s what they interact with all the time, you know, electronic game sites being problem based thinking when they’re playing games, etc, etc.  [X] It was the delivery that just made it, well a larger stumbling block, so yeah, you know I think you, if we can employ some of this technology, that sounds really exciting what you’re talking about there *** and all the others and I think that might well be the, you know, the way forward, because also what I was thinking about, you were discussing it there, was you know, what you’ve outlined here is really useful and interesting and it does have certainly, you know, it makes sense in terms of what we’d want graduates to have an understanding and be confident and competent with. | Participants like that the model brings together useful existing information and resources e.g *competency frameworks* etc ..so as *not to reinvent the wheel*. Participants like that this this is in one place for ease of access and visibility.  Participants like that the model emphasises  Involving stakeholders in different ways. *Great idea to Involve stakeholders* .. esp local chronic pain services but also *Motivational interviewing and role play amongst themselves to show how difficult it is.*  Participants like that the model gives students an ‘active’ role in pain education *the role that students play themselves*. To be active rather than passive in their learning. Participants like the interactive stuff e.g. PBL .. because it switches the focus to student problem solving? Practical pain management skills .. *the more practice people get of verbalising these very complex explanations, sometimes complex biology, complex psychology, complex sociology, that the easier it becomes for people, so that practical approach is very important.*  Participants like that the model emphasises and encourages practice of practical pain management skills e.g. *motivational interviewing*. *Virtual pain scenarios .. better than actors because it is v difficult to 'become' a patient.*  Participants like the concept of *assessing pain education* .. raises its importance, poss encourages attendance.  Participants like the use of technology to align pain education with students world. Virtual patients because they are 'real' or not ‘acting’ and technology is exciting to young people. *That’s what they interact with all the time, you know, electronic game sites being problem based thinking when they’re playing games, etc, etc.* | - Bringing everything together into one place - Give students an ‘active’ role in pain education rather than a passive learner - Switch the focus from passive theory to active problem solving learning - Emphasise practicing the practical skills needed for pain management e.g. explaining pain - Make pain education interesting and exciting with technology - The complex biology, psychology, sociology – reflect ‘realistic experiences’ - diversity | Be creative – engage students with content that requires active participation  Make pain education authentic to diverse *real* patients. Reflect realistic experiences. |
